# Prognostic value of MIB-1 proliferation index in solitary fibrous tumors of the pleura implemented in a new score – a multicenter study

**DOI:** 10.1186/s12931-017-0693-8

**Published:** 2017-12-16

**Authors:** Matthias Diebold, Alex Soltermann, Selma Hottinger, Sarah R. Haile, Lukas Bubendorf, Paul Komminoth, Wolfram Jochum, Rainer Grobholz, Dirk Theegarten, Sabina Berezowska, Kaid Darwiche, Filiz Oezkan, Malcolm Kohler, Daniel P. Franzen

**Affiliations:** 1 Department of Pulmonology, University Hospital Zurich, Raemistrasse 100, 8091 Zurich, Switzerland; 2 Institute of Pathology and Molecular Pathology, University Hospital Zurich, Zurich, Switzerland; 3 Institute of Epidemiology, Biostatistics and Prevention, University of Zurich, Zurich, Switzerland; 4 Institute of Pathology, University Hospital Basel, Basel, Switzerland; 5 Institute of Pathology, Triemli City Hospital, Zurich, Switzerland; 6 Institute of Pathology, Cantonal Hospital St. Gallen, St. Gallen, Switzerland; 7 Department of Pathology, Cantonal Hospital Aarau, Aarau, Switzerland; 8 Institute of Pathology, University Hospital Essen, University of Duisburg-Essen, Essen, Germany; 9 Institute of Pathology, University of Bern, Bern, Switzerland; 10 Department of Interventional Pulmonology, Ruhrlandklinik, University Hospital Essen, Essen, Germany; 11 James Thoracic Oncology Center, Ohio State University, Columbus, OH USA

**Keywords:** Solitary fibrous tumor, Pleura, MIB-1 proliferation index, Outcome, Score

## Abstract

**Background:**

Although the majority of solitary fibrous tumors of the pleura (SFTP) follow a benign course, 10–25% of patients suffer from recurrence or metastatic disease. Several scoring models have been proposed to predict the outcome. However, none of these included immunohistochemical (IHC) markers as possible prognosticators.

**Methods:**

In this multicenter study, we collected clinical data and formalin-fixed and paraffin-embedded (FFPE) tissue blocks of patients with histologically proven SFTP which had been surgically resected between 2000 und 2015. After systematic and extensive IHC staining on tissue microarrays, the results were analyzed and compared to histomorphological and clinical data for their possible prognostic value.

**Results:**

In total, 78 patients (mean age 61 ± 11 years) were included. Of these, 9 patients (11%) had an adverse outcome including SFTP recurrence (*n* = 6) or SFTP-related death (*n* = 3). Mean overall survival was 172 ± 13 months. 1 and 10-year event-free survival rates were 99% and 93%. In the multivariable analysis only MIB-1 proliferation index (Ki-67) ≥10% (HR 12.3, CI 1.1–139.5, *p* = 0.043), ≥4 mitoses per 10 high power fields (HR 36.5, CI 1.2–1103.7, *p* = 0.039) and tumor size larger than 10 cm (HR 81.8, CI 1.7–4016.8, *p* = 0.027) were independently associated with adverse outcome.

**Conclusion:**

A high proliferation rate by MIB-1 IHC was associated with impaired outcome. Upon this, we established a new score using mitosis, necrosis, size of the tumor and MIB-1, which performed better than the traditional scores in our data set. This prognostic score could help to better evaluate outcome of SFTP, but requires external validation.

## Background

Solitary fibrous tumors of the pleura (SFTP) are rare neoplasms of the chest cavity with an estimated incidence of 2.8 new cases/100,000 individuals/year [1, 2]. They are responsible for less than 5 % of all tumors arising from the pleura, but have been reported to occur in other anatomical locations [3–5]. Klemperer et al. were the first to describe SFTP in 1931, and since then only 900 cases have been reported up to 2005 [1, 6].

According to their distinct histological pattern, SFTP used to be called ‘hemiangiopericytoma’. A variety of other names were used, which made it difficult to classify them into one entity [7]. Histologically, they typically show alternate areas with varying hypercellularity. Characteristic in those less cellular areas is a dense collagenous background with variable assembled spindle cells, the so-called “patternless pattern” and haemangiopericytoma-like branching blood vessels. By using immunohistochemical (IHC) staining including CD34 and CD99, it has been shown that SFTP seem to originate from mesenchymal tissue [8, 9]. However, the major breakthrough in the firm diagnosis of SFTP was the detection of the NAB-STAT6 gene fusion which is present in up to 100% of these tumors and, thus, has been shown to help diagnosing these rare neoplasms and distinguish them from histologic mimics [10–12]. Furthermore, some studies suggested that a certain fusion type might be associated with different variations of SFTP [13, 14].

The majority of SFTP seem to follow a benign behavior. However, 10–25% can relapse or present signs of malignancy, i.e. metastases [15]. In 1989, England et al. proposed morphological and histological characteristics to distinguish malignant from benign variants of SFTP [16]. These included tumor size, atypical localization, sessile rather than pedunculated tumor, necrosis or hemorrhage, more than four mitoses per 10 high power fields (HPF), and nuclear pleomorphism. These features built the basis for subsequent scoring systems which have been introduced to better predict the biological behavior of SFTP [17, 18]. However, these scoring systems do not consider IHC markers as possible prognosticators of the biological behavior. The aim of this study was to investigate the role of clinical, morphological and, in particular, IHC staining as possible prognosticator of the outcome in patients with SFTP.

## Methods

### Subjects and data

Patients were recruited at seven hospitals in Switzerland and Germany. All patients with a histologically proven SFTP who were diagnosed and operated between 2000 and 2015 at one of these centers and had a follow-up of at least one month were eligible for the study. Patients’ clinical and follow-up data were drawn from the medical records and, if required, obtained from their general practitioners.

Formalin-fixed and paraffin-embedded (FFPE) tissue blocks and representative slides of every eligible patient were collected from all participating hospitals. Accuracy of the SFTP diagnosis was achieved using IHC for STAT6 c-terminus epitope as a surrogate marker for the NAB-STAT6 fusion protein which was performed in every sample. All NAB-STAT6 negative tumors were microscopically re-examined by an expert lung pathologist (A.S.). Patients with incorrect diagnosis of SFTP or missing FFPE tissue block were excluded from the study.

Written informed consent was obtained from all patients or their relatives. The study was approved by the Ethics committee of the Canton of Zurich (KEK-ZH 2012–0279), and the study was notified at ClinicalTrials.gov (Identifier: NCT01694654).

### Hybrid cytology/tissue microarray

To reproduce a representative profile of the whole tumor we obtained four cores for every patient and selected two less proliferative and two more proliferative areas of the parrafin block. From each of these regions of the donor block, a paraffin core of 0.6 mm diameter and 3–4 mm height was taken and precisely arrayed into a new recipient paraffin block using a custom-made, semiautomatic tissue arrayer (taucherinsel-neuenburg@t-online.de). Of these, 2.5 μm sections were cut for IHC staining which was performed under standardized conditions. Commercially available antibodies were used for IHC staining. The following antibodies were used: MIB-1 (Rabbit anti-Ki-67, mib 1, clone 30–9, Ventana-Roche, Tucson, AZ, USA), CD 34 (Confirm anti-CD34, clone QBEnd/10, Ventana-Roche, Tucson, AZ, USA), bcl-2 (Mouse anti-bcl-2, clone 124 Ventana-Roche, Tucson, AZ, USA), CD99 (Mouse anti-human CD99, clone HO36–1.1, Novocastra laboratories Ltd., Newcastle upon Tyne, UK), p53 (Mouse anti-human p53 Protein, clone DO-7, DAKO A/S, Glostrup, Denmark), Vimentin (Mouse anti-human Vimentin, clone VIM3B4, DAKO A/S, Glostrup, Denmark), Insulin-like-growth-factor IGF2 (Mouse anti-Insulin like growth factor 2, Cell Signaling Technology, Cambridge, UK) and NAB- STAT-6 (Stat6 S-20, Santa Cruz Biotechnology Inc., Dallas, TX, USA).

### Immunohistochemistry

Immunoreactivity of the samples was scored visually by M.D. and a dedicated pathology technician. The staining of all IHC markers except MIB-1 were semi-quantitatively scored from 0 (negative) to 3 (strong reaction) in all four cores. Finally, the mean of these scores was used for statistical analysis. For MIB-1 proliferation index, all the cells were counted visually and the percentage of cells having any positivity was calculated. The mean percentage of positive cells of all four cores was used for statistical analysis.

### Definition of adverse outcome

Adverse outcome was defined as recurrence of SFTP, development of metastases or SFTP-related death. Thus, event-free survival time was defined as the time between surgery and the occurrence of an adverse outcome.

### Statistical analysis

All statistical analyses were performed using SPSS Statistics for Windows, version 20.0 (IBM Corporation, Armonk, NY). χ2-test (categorical data) and Mann-Whitney U test (continuous data) were used to determine a significant association between the variables and an adverse outcome. Overall-survival and event-free survival were calculated using Kaplan-Meier curves. To estimate a prognostic value of each variable we used the Cox regression model. Each variable with a *p*-value less than 0.1 was entered in a multivariable analysis, and the hazard ratio (HR) and confidence interval (CI) were computed. A p-value less than 0.05 was considered to be significant. We tested morphological, clinical and IHC staining variables for their possible prognostic value. Furthermore, sensitivity and specificity of the currently used scores were computed.

## Results

### Demographics and clinical presentation

**Fig. 1:**
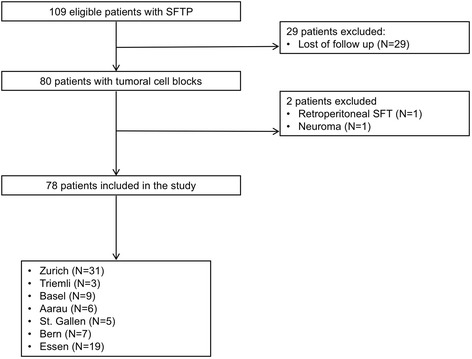
Study flowchart. SFT(P), solitary fibrous tumor (of the pleura)

From 109 eligible patients, a total of 78 patients (41% males, mean age 61 ± 11 years) were included in the study (Fig. 1). Twenty-five patients (32%) were active or former smokers. At the time of diagnosis 27 patients (35%) reported symptoms such as dyspnea (19%) and chest pain (13%). Symptomatic hypoglycemia was found in 3 patients (7%). In 32 cases SFTP was an incidental finding (41%).

### Therapy

All patients were treated surgically. SFTP were removed by video-assisted thoracoscopy (50%) or thoracotomy (50%), performing a wedge resection (82%) a lobectomy (12%) or a pneumonectomy (6%). Perioperative complications were reported in a total of 11 cases (14%) including bleeding (*n* = 1), hydrothorax (*n* = 1), prolonged air leak (*n* = 3), allergic reaction (*n* = 1), intercostal neuralgia (*n* = 1) and sepsis (*n* = 1). 30-day mortality was 0%. The median length of hospital stay was 6 (range 3–25) days.

### Event-free survival and adverse outcome

The mean overall survival after surgery was 172 ± 13 months. The mean event-free survival was 165 (±15) months with a median follow-up time of 36 months (range 1–216). After one and ten years, the event-free survival rates were 99% and 93%, respectively. Nine patients (11%) had an adverse outcome including SFTP recurrence (*n* = 6, 7.7%) and SFTP-related death (*n* = 3, 0.04%). No patient developed metastases. Another five patients died of unrelated causes during follow-up time.

### Conventional prognosticators of adverse outcome

The classical histomorphological characteristics proposed by England et al. [16] and Tapias et al. [17] are summarized in Table 1. Three of the conventional England et al. [16] features were associated with adverse outcome (Table 3). In multivariable analysis, only tumor size ≥10 cm and ≥ four mitoses/10 HPF were independently associated with adverse outcome. All the other histomorphological variables including presence of necrosis or hemorrhage, atypical location and sessile morphology were not significant. Sensitivity and specificity for the diagnosis of a malignant SFTP variant was 89% (95% CI 51.8–99.7) and 59% (95% CI 47–71), respectively, when using the characteristics by England et al. [16] (HR 18.8, 95% CI 2.2–160.6, *p* = 0.007). When applying the criteria by de Perrot et al. [18] in our cohort, the distribution of tumor stages was as follows: 17 patients stage 0 (21.3%), 6 patients stage I (7.7%), 13 patients stage II (16.7%), 8 patients stage III (10.3%) and 0 patients stage IV. Due to missing data we could not perform the score in the remaining 34 patients (43.6%). Sensitivity and Specificity for the diagnosis of a malignant SFTP variant was 100% (95% CI 29.2–100) and 56% (95% CI 39.8–71.5), respectively. Stage II and Stage III tumors were not associated with adverse outcome (HR 79.6, 95% CI 0.007–920,630, *p* = 0.359). The recently introduced score by Tapias et al. [17] which has been proposed to predict the risk of recurrence did not show a significant association between a score greater than three points and recurrence (HR 777.8, 95% CI 0.1–164,317,352, *p* = 0.287). However, sensitivity and specificity of the Tapias score for prediction of recurrence was 100% (95% CI 48–100%) and 76% (95%CI 65–86%), respectively.

**Table 1:** Histomorphological features of solitary fibrous tumors of the pleura

Characteristics	All (*n* = 78)	Adverse outcome (*n* = 9)	Favorable outcome (*n* = 69)	*p*-value*
Atypical location (parietal), n (%)	15 (19.2)	1 (12.5)	14 (20.3)	0.5
Tumor size, median (IQR)	8.2 (4.05–14.0)	17.0 (9.1–21.5)	6.3 (3.9–12.6)	0.007
Tumor size >10 cm, n (%)	31 (39.7)	7 (77.8)	24 (35.3)	0.019
Non-pedunculated, n (%)	14 (31.8)	2 (66.7)	12 (29.3)	0.2
Necrosis, n (%)	22 (28.6)	5 (62.5)	17 (24.6)	0.039
Hemorrhage, n (%)	17 (22.1)	3 (37.5)	14 (20.3)	0.4
Mitotic count, median (IQR)	1 (1.0–3.75)	7 (3–15)	1 (1–3)	0.005
> 4 mitoses / 10 HPF, n (%)	16 (25)	5 (71.4)	11 (19.3)	0.009
Pleomorphism, n (%)	8 (10.4)	3 (37.5)	5 (7.2)	0.033
Hypercellularity	19 (57.6)	7 (87.5)	12 (48)	0.056

*HPF* high power field, *IQR* interquatile range

*Chi-square test or Mann-Whitney U-test as appropriate

### Immunohistochemical markers as possible prognosticators of adverse outcome

The IHC staining profile of SFTP is displayed in Table 2. For MIB-1 proliferation index scores ranged from 0 to 50% with a median of 1 (IQR 1–3). When comparing the HRs and *p*-values of MIB-1 proliferation index in univariable analysis a cut-off value of 10% performed best to predict adverse outcome. In the univariable analysis, only MIB-1 proliferation index (Ki-67) ≥10% was significantly associated with adverse outcome.

**Table 2:** Immunohistochemical staining profile of solitary fibrous tumors of the pleura

Staining	All (*n* = 78)	Adverse outcome (*n* = 9)	Favorable outcome (*n* = 69)	*p*-value*
CD34, n (%)				0.6
0	0 (0)	0 (0)	0 (0)	
1	11 (14.1)	2 (22.2)	9 (13.0)	
2	27 (34.6)	2 (22.2)	25 (36.2)	
3	40 (51.3)	5 (55.6)	35 (50.7)	
Bcl-2, n (%)				0.2
0	0 (0)	0 (0)	0 (0)	
1	25 (32.1)	3 (33.3)	22 (31.9)	
2	27 (34.6)	1 (11.1)	26 (37.7)	
3	26 (33.3)	5 (55.6)	21 (30.4)	
Vimentin, n (%)				n/a
0	0 (0)			
1	0 (0)			
2	0 (0)			
3	78 (100)			
NAB-STAT6, n (%)				0.86
0	3 (3.8)	2 (22.2)	1 (1.4)	
1	24 (30.8)	1 (11.1)	23 (33.3)	
2	35 (44.9)	3 (33.3)	32 (46.4)	
3	16 (20.5)	3 (33.3)	13 (18.8)	
p53, n (%)				0.13
0	3 (3.9)	0 (0)	3 (4.5)	
1	40 (52.6)	4 (44.4)	36 (53.7)	
2	28 (36.8)	2 (22.2)	26 (38.8)	
3	5 (6.6)	3 (33.3)	2 (3)	
IGF2, n (%)				n/a
0	0 (0)			
1	0 (0)			
2	0 (0)			
3	78 (100)			
CD99, n (%)				0.6
0	4 (5.1)	1 (11.1)	3 (4.3)	
1	39 (50.0)	5 (55.6)	34 (49.3)	
2	22 (28.2)	1 (11.1)	21 (30.4)	
3	13 (16.7)	2 (22.2)	11 (15.9)	
MIB-1 proliferation index (Ki-67), median % (IQR)	1 (1.0–3.0)	10 (1–17)	1.0 (1.0–2.5)	0.044
MIB-1 proliferation index (Ki-67) ≥ 10%, n (%)	5 (6.4)	5 (55.6)	0 (0)	< 0.001

The stainings were classified as negative (0), weak (1), intermediate (2) and strong (3)

*HPF* high-power field, *n/a* not applicable

*Chi-square test or Mann-Whitney U-test as appropriate

**Fig. 2:**
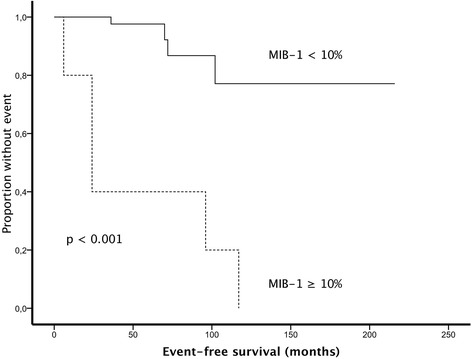
Kaplan-Meier survival curve according to MIB-1 proliferation index (Ki-67)

MIB-1 proliferation index remained significant in multivariable analysis (HR 13.3, CI 3.5–50.7, *p* = 0.043) (Table 3). Kaplan-Meier survival curve according to MIB-1 proliferation index is shown in Fig. 2.

**Table 3:** Univariable and multivariable analysis of possible predictors of the biological behavior of solitary fibrous tumors of the pleura

Variable	Univariable analysis		Multivariable analysis	
	HR (95% CI)	*p*-value	HR (95% CI)	*p*-value
Tumor size ≥10 cm	8.8 (1.8–43.5)	0.008	81.8 (1.7–4016.8)	0.027
Necrosis	3.4 (0.8–14.6)	0.089	1.7 (0.1–18.4)	0.7
MIB-1 ≥ 10%	13.3 (3.6–50.7)	< 0.001	12.3 (1.1–139.5)	0.043
Mitoses ≥4/10 HPF	13.1 (1.6–112.0)	0.019	36.6 (1.2–1103.7)	0.039
Atypical location	1.6 (0.2–13.4)	0.641		
Non-pedunculated	196.6 (0.0–126,601,532.0)	0.439		
Pleomorphism	9.8 (2.2–44.5)	0.003	11.2 (0.6–22.3)	0.1

### Proposal of a new scoring system

**Fig. 3:**
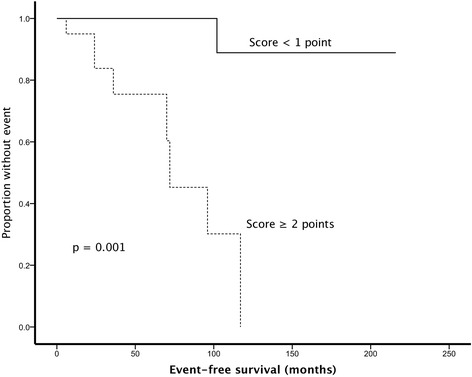
Kaplan-Meier survival curve according the new scoring system

On the basis of the above data, we established a new scoring system containing four variables, attributing to one point for each variable if it was above a specific threshold (Table 4). If a variable was missing, it was assumed to be less than the threshold and assigned a point value of 0. For the proposed scoring system, we included all three variables, which remained significant in the multivariable analysis in addition to presence of necrosis, which is one well-validated risk criteria as shown in Table 4.34 patients had a score of 0 (43.6%), 24 patients a score of 1 (30.8%), 12 patients a score of 2 (15.3%), 6 patients a score of 3 (7.7%) and two patients had a score of 4 (2.6%). When using a score of two or more point, the score performed best and was significantly associated with adverse outcome (HR 32.9, CI 4.0–270.9, *p* = 0.001). Kaplan-Meier survival curve according to the new score is shown in Fig. 3. Sensitivity and specificity was 89% (95% CI 52–100%) and 83% (95%CI 72–91%), respectively.

**Table 4:** Proposal of a new scoring system for solitary fibrous tumors of the pleura

Variables	Points
Mitoses ≥4/10 HPF	1
Mitoses <4/10 HPF (or Mitoses missing)	0
Tumor size ≥10 cm	1
Tumor size <10 cm (or tumor size missing)	0
MIB-1 ≥ 10%	1
MIB-1 < 10% (or MIB-1 missing)	0
Necrosis	1
No Necrosis (or necrosis missing)	0

Score interpretation: Each variable scoring a point with a maximum of four points and a minimum of zero points. A missing variable was computed as a value of 0

A score of ≥1 point was associated with adverse outcome with a sensitivity of 88% (95% CI 52–100%) and a specificity of 48% (95% CI 36–60%). (HR 7.03, CI 0.87–56.69, *p* = 0.067)

A score of ≥2 points was associated with adverse outcome with a sensitivity of 89% (95% CI 52–100%) and a specificity of 83% (95% CI 72–91%). (HR 32.9, CI 4.0–270.9, *p* = 0.001)

A score of ≥3 points was associated with adverse outcome with a sensitivity of 33% (95% CI 7–70%) and a specificity of 94% (95% CI 86–98%). (HR 4.6, CI 1.2–18.5, *p* = 0.031)

A score of 4 points was associated with adverse outcome with a sensitivity of 22% (95% CI 3–60%) and a specificity of 100% (95% CI 95–100%). (HR 102.03, CI 8.97–1160.95, *p* < 0.001)

## Discussion

In this multicenter study, we collected clinical data and FFPE tissue blocks of patients with histologically proven SFTP, aiming to investigate IHC markers which may improve predicting the biological behavior of SFTP. Eleven percent of patients had an adverse outcome. SFTP-related lethality was 0.04%, whereas recurrence rate was 7.7% compared to 10–25% in other series [15, 16, 18]. This could be related to a shorter follow-up time in our study with a median follow-up time of 36 months compared to a median follow-up time of 70 months in the study of Schirosi et al. [15].

While the majority of SFTP follow a benign course, few SFTP will behave aggressively and lead to recurrence, tumor-related death or metastatic disease. However, many SFTP originally classified as malignant follow a benign course, and vice-versa [16, 19]. Therefore, England et al. proposed six histomorphological features to characterize malignant variants [16]. These features are widely accepted and used in many centers. It is not clearly defined though, which and how many of these features have to be fulfilled to define a malignant variant of SFTP. In our study, from the variables included in the criteria proposed by England et al., [16] only tumor size greater than 10 cm, nuclear pleomorphism and increased mitotic activity (more than four mitoses per 10 HPF) were significantly associated with an adverse outcome in univariable analysis. Yet, in the multivariable analysis, only two of these features (mitotic count and tumor diameter) remained significant. This finding was already confirmed by other studies demonstrating that only mitotic count and tumor size were associated with impaired outcome [20, 21]. Furthermore, the specificity of the England criteria was challenged by Schirosi et al. who showed that at least one of these variables is present in 59% of SFTP, whereas mortality and recurrence rate are only 10% and 18%, respectively [15].

Recently new scoring systems have been proposed to predict either the risk of recurrence or the combined risk of adverse outcome, namely recurrence, development of metastasis or SFTP-related death [17, 18]. De Perrot et al. proposed a model based on morphologic and histologic features (morphology, high cellularity, cellular pleomorphism, high mitotic count, necrosis or stromal/vascular invasion) with a treatment plan and recommendation of adjuvant therapy and follow up [18]. Likewise, Tapias et al. suggested a scoring system to predict the risk of recurrence based on certain histologic and morphologic features (pleural origin, morphology, size, hypercellularity, presence of necrosis or haemorrhage, number of mitosis per 10 HPFs) [17]. However, we could not confirm those scores proposed by Tapias et al. [17] in our cohort, since it was not associated with the risk of recurrence or adverse outcome.

In the end, none of the grading systems based on histomorphological features is sufficiently accurate to predict the biological behavior of SFTP at the time of diagnosis. Thus, attempts to find new biomarkers or to expand the conventional grading systems by new biomarkers is warranted. However, none of the above mentioned studies investigated the prognostic value of IHC markers. A reason for this could be that IHC staining for SFTP is not standardized and, thus, not uniformly used in different centers. Likewise, MIB-1 proliferation index (Ki-67) has not been routinely measured, although some studies showed a trend towards a promising prognostic value of MIB-1 [17, 20, 22]. We collected FFPE tissue blocks of patients with histologically proven SFTP from seven hospitals in two countries and uniformly performed IHC staining on them. Loss of CD34 expression has been shown to be more prevalent in malignant SFTP [23]. We could not confirm this finding, since all SFTP in our study were positive for CD34. Furthermore, positivity of the tumor suppressor protein p53 has been shown to be associated with malignant features [15, 23]. In our study, p53 expression was not significantly more prevalent in patients with adverse outcome. NAB-STAT6 gene fusion has been shown to be present in the vast majority of SFTP and is used for diagnosis of these tumors with a sensitivity and specificity of 98% and 85%, respectively [10–12, 24]. In our study only three SFTP showed negativity for NAB-STAT6 resulting in a sensitivity of 96% in diagnosing SFTP, which is similar to other published series [10, 11]. MIB-1 is an antibody against Ki-67 which is a protein associated with cell proliferation and ribosomal RNA synthesis. Thus, MIB-1 is a reliable IHC marker of cell proliferation, and MIB-1 proliferations index (Ki-67 expression) is associated with poor prognosis in various cancers, especially breast and prostate cancer [25, 26]. However, the prognostic value of MIB-1 proliferation index has not yet been systematically investigated in SFTP. To date, there is only one retrospective study in a relatively small cohort published by our group, revealing that a high proliferation index assessed with Ki-67 was associated with poor prognosis [20]. To the best of our knowledge, the present study is the largest to systematically investigate the role of MIB-1 in SFTP. We found that a high proliferation index MIB-1 of more than 10% was independently associated with adverse outcome.

Upon this finding, we established a new score using the variables which have been shown to correlate with impaired outcome. This score, using mitosis count, tumor size, MIB-1 and necrosis performed better than traditional scores in predicting adverse outcome of SFTP in our cohort. Compared to the criteria proposed by England et al. [16] or the score proposed by Tapias et al. [17] this score is the first to use an IHC marker. We excluded histomorphological features, such as pleomorphism, nuclear atypia and hemorrhage, since they are subject to inter-observer variability and lack clear-cut objectiveness. Certainly, this score has to be validated in a larger population, but, nevertheless we highly recommend including MIB-1 in any future score.

A clear strength of our study is a considerably high patient number drawn from multiple centers despite the low incidence of SFTP. Furthermore, the design of the study allowed a systematical and uniform investigation of IHC staining and their prognostic value. However, some limitations merit consideration. First, there was a relatively short follow-up time. Secondly, due to lack of variety of proliferation rates we could not perform a receiver operating curve (ROC) analysis to evaluate the best cut-off value of MIB-1. Finally, considering the histological structure of SFTP with alternate areas of more or less proliferation, it has to be further evaluated, whether our scoring of MIB-1 using two less and two more proliferative areas is reproducible in other series.

## Conclusion

In addition to tumor diameter and mitotic count, MIB-1 proliferation index is a significant prognosticator of adverse outcome in patients with SFTP. A prognostic score including these three features and necrosis performed better than the traditional scores only based on histomorphological criteria. Validation of this score is recommended in a future study with longer follow-up time.

## References

[1] Chick JF, Chauhan NR, Madan R (2013). Solitary fibrous tumors of the thorax: nomenclature, epidemiology, radiologic and pathologic findings, differential diagnoses, and management. AJR Am J Roentgenol.

[2] Briselli M, Mark EJ, Dickersin GR (1981). Solitary fibrous tumors of the pleura: eight new cases and review of 360 cases in the literature. Cancer.

[3] Okike N, Bernatz PE, Woolner LB (1978). Localized mesothelioma of the pleura: benign and malignant variants. J Thorac Cardiovasc Surg.

[4] Zhanlong M, Haibin S, Xiangshan F, Jiacheng S, Yicheng N (2016). Variable solitary fibrous tumor locations: CT and MR imaging features. Medicine (Baltimore).

[5] Gengler C, Guillou L (2006). Solitary fibrous tumour and haemangiopericytoma: evolution of a concept. Histopathology.

[6] Klemperer P, Coleman BR (1992). Primary neoplasms of the pleura. A report of five cases. Am J Ind Med.

[7] Stout AP, Murray MR (1942). Hemangiopericytoma: A vascular tumor featuring zimmermann's pericytes. Ann Surg.

[8] el-Naggar AK, Ro JY, Ayala AG, Ward R, Ordóñez NG (1989). Localized fibrous tumor of the serosal cavities. Immunohistochemical, electron-microscopic, and flow-cytometric DNA study. Am J Clin Pathol.

[9] Flint A, Weiss SW (1995). CD-34 and keratin expression distinguishes solitary fibrous tumor (fibrous mesothelioma) of pleura from desmoplastic mesothelioma. Hum Pathol.

[10] Chmielecki J, Crago AM, Rosenberg M, O'Connor R, Walker SR, Ambrogio L, Auclair D, McKenna A, Heinrich MC, Frank DA, Meyerson M (2013). Whole-exome sequencing identifies a recurrent NAB2-STAT6 fusion in solitary fibrous tumors. Nat Genet.

[11] Robinson DR, Wu YM, Kalyana-Sundaram S, Cao X, Lonigro RJ, Sung YS, Chen CL, Zhang L, Wang R, Su F (2013). Identification of recurrent NAB2-STAT6 gene fusions in solitary fibrous tumor by integrative sequencing. Nat Genet.

[12] Doyle LA, Vivero M, Fletcher CD, Mertens F, Hornick JL (2014). Nuclear expression of STAT6 distinguishes solitary fibrous tumor from histologic mimics. Mod Pathol.

[13] Tai HC, Chuang IC, Chen TC, Li CF, Huang SC, Kao YC, Lin PC, Tsai JW, Lan J, Yu SC (2015). NAB2-STAT6 fusion types account for clinicopathological variations in solitary fibrous tumors. Mod Pathol.

[14] Barthelmeß S, Geddert H, Boltze C, Moskalev EA, Bieg M, Sirbu H, Brors B, Wiemann S, Hartmann A, Agaimy A, Haller F (2014). Solitary fibrous tumors/hemangiopericytomas with different variants of the NAB2-STAT6 gene fusion are characterized by specific histomorphology and distinct clinicopathological features. Am J Pathol.

[15] Schirosi L, Lantuejoul S, Cavazza A, Murer B, Yves Brichon P, Migaldi M, Sartori G, Sgambato A, Rossi G (2008). Pleuro-pulmonary solitary fibrous tumors: a clinicopathologic, immunohistochemical, and molecular study of 88 cases confirming the prognostic value of de Perrot staging system and p53 expression, and evaluating the role of c-kit, BRAF, PDGFRs (alpha/beta), c-met, and EGFR. Am J Surg Pathol.

[16] England DM, Hochholzer L, McCarthy MJ (1989). Localized benign and malignant fibrous tumors of the pleura. A clinicopathologic review of 223 cases. Am J Surg Pathol.

[17] Tapias LF, Mino-Kenudson M, Lee H, Wright C, Gaissert HA, Wain JC, Mathisen DJ, Lanuti M (2013). Risk factor analysis for the recurrence of resected solitary fibrous tumours of the pleura: a 33-year experience and proposal for a scoring system. Eur J Cardiothorac Surg.

[18] de Perrot M, Fischer S, Brundler MA, Sekine Y, Keshavjee S (2002). Solitary fibrous tumors of the pleura. Ann Thorac Surg.

[19] Vallat-Decouvelaere AV, Dry SM, Fletcher CD (1998). Atypical and malignant solitary fibrous tumors in extrathoracic locations: evidence of their comparability to intra-thoracic tumors. Am J Surg Pathol.

[20] Franzen D, Diebold M, Soltermann A, Schneiter D, Kestenholz P, Stahel R, Weder W, Kohler M (2014). Determinants of outcome of solitary fibrous tumors of the pleura: an observational cohort study. BMC Pulm Med.

[21] van Houdt WJ, Westerveld CM, Vrijenhoek JE, van Gorp J, van Coevorden F, Verhoef C, van Dalen T (2013). Prognosis of solitary fibrous tumors: a multicenter study. Ann Surg Oncol.

[22] Hiraoka K, Morikawa T, Ohbuchi T, Katoh H (2003). Solitary fibrous tumors of the pleura: clinicopathological and immunohistochemical examination. Interact Cardiovasc Thorac Surg.

[23] Yokoi T, Tsuzuki T, Yatabe Y, Suzuki M, Kurumaya H, Koshikawa T, Kuhara H, Kuroda M, Nakamura N, Nakatani Y, Kakudo K (1998). Solitary fibrous tumour: significance of p53 and CD34 immunoreactivity in its malignant transformation. Histopathology.

[24] Demicco EG, Harms PW, Patel RM, Smith SC, Ingram D, Torres K, Carskadon SL, Camelo-Piragua S, McHugh JB, Siddiqui J (2015). Extensive survey of STAT6 expression in a large series of mesenchymal tumors. Am J Clin Pathol.

[25] Luporsi E, André F, Spyratos F, Martin PM, Jacquemier J, Penault-Llorca F, Tubiana-Mathieu N, Sigal-Zafrani B, Arnould L, Gompel A (2012). Ki-67: level of evidence and methodological considerations for its role in the clinical management of breast cancer: analytical and critical review. Breast Cancer Res Treat.

[26] Tollefson MK, Karnes RJ, Kwon ED, Lohse CM, Rangel LJ, Mynderse LA, Cheville JC, Sebo TJ (2014). Prostate cancer Ki-67 (MIB-1) expression, perineural invasion, and gleason score as biopsy-based predictors of prostate cancer mortality: the Mayo model. Mayo Clin Proc.

